# Genomic epidemiology of strains currently and formerly classified as *Enterobacter* spp. recovered from equine necropsy samples

**DOI:** 10.1371/journal.pone.0333701

**Published:** 2025-11-13

**Authors:** Blandine Harel, Corinne Sévin, Simon Le Hello, Peggy Moreau, Jean-Christophe Giard, Sandrine Petry, François Gravey

**Affiliations:** 1 Université de Caen Normandie, Université de Rouen Normandie, INSERM, DYNAMICURE UMR1311, F-14000 Caen, France; 2 ANSES, Normandy Laboratory for Animal Health, Physiopathology and Epidemiology of Equine Diseases Unit, Goustranville, France; 3 Department of Infectious Agents, Bacteriology, CHU Caen, F-14000 Caen, France; Tianjin University, CHINA

## Abstract

*Enterobacteriaceae* are opportunistic pathogens responsible for local or systemic infections in both human and veterinary medicine. To monitor circulating strains in stud farms in Normandy (France), we investigated a collection of *Enterobacteriaceae* isolated from necropsied equids performed in the region between 1997 and 2020. These strains were initially identified using MALDI-TOF; however, as this method failed to identify some isolates, whole genome sequencing followed by rMLST analysis was subsequently performed. Different genera were identified: *Enterobacter* spp., *Huaxiibacter* spp., *Lelliottia* spp., *Rahnella* spp.. MALDI-TOF and rMLST identifications were concordant for only 26.5% of the strains studied, leading us to conclude that rMLST is a more reliable method for both genus- and species-level identification, particularly for less-studied genera such as *Huaxiibacter* spp. and *Rahnella* spp.. The genus *Enterobacter* spp. (*E. hormaechei* and *E. ludwigii*) accounted for 53% of the strains with a high degree of sequence type (ST) diversity. These include *E. hormaechei* ST114 and ST171, known as high-risk clone in human clinical medicine. These clones, containing plasmids and acquired resistance genes such as *blaOXA-1*, *blaSHV-12* or *blaTEM-1B*, are resistant to at least four classes of antibiotics. The presence of genes encoding the enteroaggregative heat-stable enterotoxin 1 or the bacteriocin colicin, probably carried by plasmids, implies that *Enterobacter* spp. form a reservoir of antibiotic resistance and virulence factors. Conversely, strains of the genera *Huaxiibacter* spp., *Lelliottia* spp. and *Rahnella* spp. naturally found in the environment, showed a lean resistome and virulome. This analysis shows that genomic studies are essential to obtain precise species identification, monitor and detect high-risk clones, and to highlight the circulation of resistance and virulence genes through mobile genetic elements.

## Introduction

Horses have their specific bacterial pathogens, such as *Streptococcus equi* subspecies *equi* and *Taylorella equigenitalis*, responsible for strangles and contagious equine metritis, respectively [1]. Some infections can be caused by pathogens found also in human medicine, such as *Pseudomonas aeruginosa* and *Enterobacteriaceae*, in particular those resistant and/or virulent ones [2–5]. *Enterobacter* spp. is a ubiquitous Gram-negative bacillus, found in a variety of human clinical infections, especially in intensive care unit, on land and water, as well as in insects and animals; it is also considered as a phytopathogen [6]. The *Enterobacter* genus, firstly described in 1960, comprises 22 species, including six that form the *Enterobacter cloacae* complex (ECC)—*E. cloacae*, *E. asburiae*, *E. hormaechei*, *E. kobei*, *E. ludwigii*, *E. mori*, and *E. nimipressuralis* [7]—which are the most commonly found in human infections [8]. The six species of the ECC share at least 60% genomic homology and are divided into 12 clusters (based on average nucleotide identity analysis and *hsp60* sequencing) and 18 clades [7–9]. Advances in sequencing technology have improved the resolution of *Enterobacter* species identification and led to reclassification of the genera *Lelliottia* spp*., Pluralibacter* spp*., Kosakonia* spp*.* and *Cronobacter* spp*.* formerly included in the *Enterobacter* genus, based on multilocus sequence analysis (MLSA) [9].

A study conducted in France from 2006 to 2016 showed that *Enterobacter* spp. accounted for 3.4% of equine bacterial infections, and was found mainly in pulmonary infections [3]. This study was continued from 2016 to 2019, and revealed that *Enterobacter* was found in 2.1% of samples suspected of causing infection in horses [10], suggesting that the prevalence of *Enterobacter* infection is stable over time and is not frequently found in equine infections.

Due to the expansion of antibiotic resistance, surveillance of circulating multidrug resistance (MDR) strains in human and veterinary medicine is crucial, especially in the equine sector, where humans and animals are in close relationship. *Enterobacter* are opportunistic pathogens that can display an MDR phenotype in particular through its ability to acquire mobile genetic elements and its adaptation to different environments [7]. Additional resistances can be acquired by plasmid encoding β-lactamases genes such as *tem, shv* and *ctx-m* [7]. Strains can also be resistant to aminoglycoisides, via the synthesis of drug-metabolizing enzymes such as N-6’-acetyltransferase-ln (*aac(6’)-Ib*) or *aac(3)-IIa* and *ant(2′′)-Ia*, which confer resistance to amikacin and gentamycin [11]. Genes such as *aac(6’)-Ib-cr* and *qnr* confer fluoroquinolone resistance by inactivating the antibiotic or protecting DNA gyrase from its action, respectively [11]. Chromosomal mutations can also be the cause of antimicrobial resistance. *Enterobacter* are naturally resistant to β-lactams notably penicillins and 1^st^ generation cephalosporin by producing the chromosomal encoded cephalosporinase *ampC* [12]. AmpC overexpression phenomena are responsible for increased resistance levels, notably through mutations in its repressor AmpD or in the regulator AmpR, which can convert it into a constitutive activator [13]. *Enterobacter* also modulates membrane permeability by modifying porin expression (OmpC) and antibiotic efflux pumps like AcrAB-TolC [7]. From 2006 to 2019, in France, a study observed some strains of *Enterobacter* spp. from equine infections resistant to streptomycin (32–64% of strains), tetracycline (37–56%) or amikacin (< 10%). In 2016, 26% of the strains were resistant to at least three classes of antibiotics (aminoglycosides, tetracycline and sulfonamide), a percentage that rises to 52% in 2019 [3,10].

Within the ECC, strains are capable of forming robust biofilms, particularly under nutrient-deficient conditions [14]. This ability is linked to their capacity to adhere to surfaces through the production of type I or III *fimbriae* (encoded by the *fimA* and *fimH*, or *mrkB* genes, respectively), as well as *curli* fibers (encoded by *csgA, csgB*, and *csgD*) [15]. *Enterobacter* spp. also possess a type VI secretion system (T6SS), which contributes to adhesion to eukaryotic cells during infection and mediates interbacterial communication within the biofilm [16]. In addition, *Enterobacter* species are motile via *flagella*-mediated swimming. Their ability to utilize siderophores also makes them highly competitive in nutrient-limited environments.

The aim of this study was to characterize the antimicrobial resistance profiles and the genomic characteristic of a collection of *Enterobacter* spp*.* found in equine necropsy specimens and suspected to be associated with the death. The genomic population structure, virulome, resistome and antimicrobial susceptibility profiles were investigated, revealing the presence of the genera *Enterobacter* spp., *Lelliottia* spp., *Huaxiibacter* spp., and *Rahnella* spp. among the necropsy samples. Notably, two *E. hormaechei* strains (ST114 and ST171), considered high-risk clones in human clinical medicine, were identified.

## Materials and methods

### Sample collection

Thirty-four strains were isolated, between 1997 and 2020, from 34 equine necropsies of unknown sex (n = 1), male (n = 15) and female horses (n = 18), either Thoroughbred (n = 23), French trotters (n = 10) or unknown (n = 1). There were fetuses and their fetal membrane (n = 21), foals (n = 8) and yearlings or adults (n = 5). Various types of samples were collected, which included allantochorion (n = 27), lung (n = 6) and uterine swab (n = 1). One strain was isolated from each equine sample. All horses came from Normandy (France), except one from Pays-de-la-Loire and one of unknown origin (S1 Table). The necropsies, sample collection and aerobic bacteriological analyses were conducted by ANSES (French Agency for Food, Environmental and occupational Health & Safety) Normandy Laboratory for Animal Health. Strains were preserved in CryoBeads (BioMérieux) at −80°C.

### Bacterial culture and phenotypical identification

Bacteria were isolated on CHROMID® CPS® Elite (CPSE) agar plates (BioMérieux) incubated 24 h at 37°C. As recommended by the manufacturer, the appearance of blue colonies indicated the presence of *Enterobacter* spp., which were initially identified using matrix-assisted laser desorption/ionization time-of-flight mass spectrometry (MALDI-TOF, Bruker Daltonik), as a first-line identification method.

### Antimicrobial susceptibility testing

Antibiotic susceptibility was tested using the disk diffusion method on Mueller-Hinton agar inoculated with a 0.5 McFarland bacterial suspension and interpreted according to CA-SFM 2024 (V1.0), following the recommendations of the European Committee on Antimicrobial Susceptibility Testing (EUCAST) (www.sfm-microbiologie.org). Thirty antibiotic discs (BioRad, Hercule, CA, USA) were tested (S2 Table). Antibiograms were read using the SirScan Orion automated system (i2a, Montpellier, France). The antibiotics selected for testing were those commonly used in human medicine, reflecting the epidemiological aim of the study within a One Health approach, rather than a veterinary diagnostic purpose.

### DNA extraction and genome sequencing

Bacteria were overnight cultured in a tryptic soy broth at 37°C for 18 h. After centrifugation, bacterial pellets were resuspended in 200 µl of PBS and lysed using Magna Pure Lysis Buffer (Roche) and Proteinase K (Roche). DNA extractions were performed on the EZ1 automate (Qiagen). DNA libraries were prepared using the DNA prep kit (Illumina) according to the manufacturer instructions and sequenced on the Nextseq 500 system (Illumina). The quality of the reads was assessed using FastQC (https://www.bioinformatics.babraham.ac.uk/projects/fastqc/) and MultiQC [17]. Then *de novo* assemblies were performed with Skesa [18,19] and controlled by Quast [20]. The 34 genomes are available on NCBI (Bioproject: PRJNA1280983).

### *In silico* analysis

Species identification and sequence type (ST) determination were performed using the rMLST tool and Multi Locus Sequence Typing method (MLST) with PubMLST website, respectively. The allelic profile of the *dnaA, fusA, gyrB, leuS, pyrG, rplB* and *rpoB* genes was determined for MLST [21]. To observe whether strains were phylogenetically close, we created a tree using Mashtree [22], which was visualized on iTol [23]. Seventeen reference genomes from various *Enterobacteriaceae* such as *Escherichia coli*, *Klebsiella pneumoniae* and *Serratia marcesens*, as well as genomes from strains closely related to those we identified (*Enterobacter* spp., *Rahnella* spp., *Huaxiibacter* spp. and *Lelliottia* spp.), were used as reference genomes (S3 Table). All the reference genomes have been downloaded with NCBI-download tool (DOI: https://doi.org/10.5281/zenodo.8192432). In order to confirm the taxonomic identification of the isolates, a genomic similarity analysis was performed using the Mash v2.3 tool. The entire genomes of the isolates and those of the corresponding reference strains were used to calculate Mash distances based on k-mer sketches (default parameters: k = 21, s = 1,000). A distance of less than 0.05 was considered indicative of belonging to the same species (corresponding to an ANI greater than 95%). The resistome and virulome were characterized by Resfinder [24] (http://genepi.food.dtu.dk/resfinder) and Virulence Factor DataBase [25] (https://www.mgc.ac.cn/VFs/), respectively. Virulence genes with an 80% identity and 70% of coverage threshold were considered. The plasmidome was determined using the PlasmidFinder database [26] (with a minimum identity of 90% and a minimum coverage of 80%) and then confirmed using the PLSDB database [27].

## Results

### Species identification

Thirty-four strains have been previously characterized by the ANSES laboratory as *Enterobacter* spp., using phenotypic methods such as Gram staining (Gram-negative *bacilli*) and API20E galleries. From the CryoBeads storage tubes containing the isolates, strains were cultured using CPSE agar plates to ensure the purity of the samples (S1 Fig). Strains exhibiting turquoise-blue colonies characteristic of *Enterobacter* spp. were initially identified using MALDI-TOF as first-line identification method, which revealed the presence of *Enterobacter* spp., *Lelliottia* spp., *Rahnella* spp. (previously classified under *Enterobacter* spp.).

For 9/34 strains (26.5%) MALDI-TOF failed to identify the species and the genus. About half of the strains (15/34) were characterized as members of the *Enterobacter* genus, including *E. cloacae* complex (n = 5)*, E. ludwigii* (n = 5), *E. cancerogenus* (n = 2)*, E. asburiae* (n = 1)*, E. hormaechei* (n = 1)*,* and *E. bugandensis* (n = 1). A quarter of the strains were characterized as belonging to the genus *Lelliottia* (8/34), with two species which belonged to thisgenus: *L. amnigena* (7/34) and *L. nimipressuralis* (1/34). MALDI-TOF also identified two *Rahnella aqualitis* strains.

**Fig 1:**
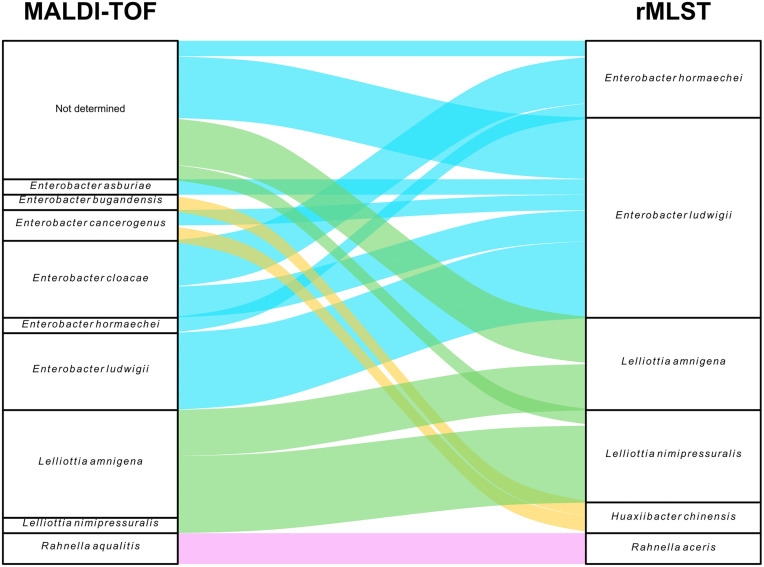
Alluvial plot of comparison of identification of equine strains by MALDI-TOF and rMLST. The same isolates were characterized by both methods. Colors were assigned based on rMLST identification: *Enterobacter* spp. (blue), *Rahnella* spp. (pink), *Lelliottia* spp. (green), and *Huaxiibacter* spp. (yellow).

**Fig 2:**
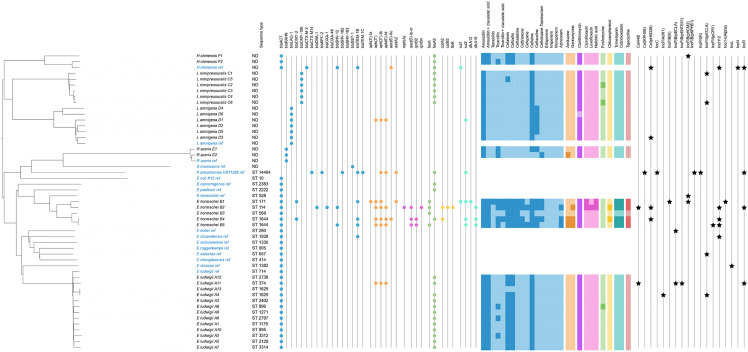
Phylogenetic tree of the collection strains associated with 17 reference genomes (in blue) downloaded from NCBI. Sequence types (STs) are determined for the *Enterobacter* spp. genus, ND corresponds to non-determined STs. Colored dots represent the presence of antibiotic resistance genes against β-lactams (blue), aminosides (orange), macrolides (violet), quinolones (pink), fosfomycines (green), phenicols (yellow) and sulfonamides (turquoise). The heatmap shows antibiotic resistance phenotypes (dark squares: resistant, light squares: susceptible).

Due to the limitations of MALDI-TOF in species-level identification (score < 2), all strains were subsequently subjected to whole-genome sequencing to achieve accurate genomic identification. Strain identification using the rMLST allowed species-level assignment for all strains. Whereas MALDI-TOF identifies nine species types, rMLST identifies six species. Fig 1 and S4 Table. shows MALDI-TOF and sequencing identification of the 34 *Enterobacter* or formerly *Enterobacter*. Both methods showed 26.5% common identifications (9/34): five *E. ludwigii*, three *L. amnigena* and one *E. hormaechei*. Two *Huaxiibacter chinensis* were only identify using the rMLST approach (Fig 1). For the genus *Enterobacter*, MALDI-TOF identified six species (*E. cancerogenus, E. asburiae, E. cloacae* complex*, E. hormaechei, E. ludwigii and E. bugandensis*), while the genomic approach revealed the presence of two species (*E. hormaechei and E. ludwigii*). Regarding the nine strains that could not be identified by MALDI-TOF, rMLST revealed that four belonged to the genus *Lelliottia* and five to the genus *Enterobacter*. As for the other scenarios observed: i) among the seven strains of *L. amnigena* identified by MALDI-TOF, three strains were confirmed by rMLST but four were characterized as *L. nimipressuralis*; ii) two strains identified as *Rahnella aqualitis* by MALDI-TOF were classified as *Rahnella asceris* by rMLST; and iii) for the genus *Enterobacter*, MALDI-TOF identified six species: *E. cancerogenus, E. asburiae, E. cloacae* complex*, E. hormaechei, E. ludwigii, and E. bugandensis*, while the rMLST revealed only two species: *E. hormaechei* (n = 5) *and E. ludwigii* (n = 13). The five ECC strains identified by MALDI-TOF corresponded to three *E. hormaechei* and two *E. ludwigii* strains, as confirmed by rMLST. MLST typing was then performed to establish the STs of *Enterobacter* spp. (Fig 2). Twelve STs were found for *E. ludwigii*: ST374, ST895, ST896, ST1179, ST1271, ST1629, ST2129, ST2402, ST2738, ST2797, ST3312, and ST3314; and four STs for *E. hormaechei*: ST114, ST171, ST68, and ST1644. ST1629 and ST1644 were each detected in two strains. For the remainder of the study, we retained WGS-based characterization, as it allows for genomic discrimination within the *Enterobacter cloacae* complex strains as well as the characterization of newly classified genera such as *Huaxiibacter* spp. The rationale for this choice is detailed in the Discussion section. To resolve discrepancies between MALDI-TOF and rMLST results, we used the Mash tool to estimate genomic similarity (Mash distance) between our isolates and reference genomes from NCBI. The lowest distances (<0.05) consistently corresponded to the species identified by rMLST and these reference genomes, confirming their taxonomic assignment (S5 Table).

Following the identification of the strains, we performed statistical tests (Fischer’s exact test) to determine whether there was a significant prevalence of certain species according to sampling period, sample type, breed (French Trotters versus Thoroughbreds), gender (male versus female), or life stage (fetus versus foal/yearling/adult). We observed that *E. hormaechei* was significantly more prevalent in horse necropsies at the “foal”, “yearling”, or “adult” stages compared to the “fetus” stage (p-value = 0.0046). We also observed that *E. hormaechei* is significantly more prevalent in samples from lungs than in other samples (p-value = 0.0017) (S6 Table).

### Phylogeny

The MashTree (Fig 2) is based on the calculation of distance based on the k-mers shared between 34 genomes of the strains in the collection and 17 reference genomes available from the NCBI database. It can be used to evaluate the genomic proximity between strains via a distance matrix. We observe that the tree is divided into two main groups. The first group comprises strains of the genera *Huaxiibacter* spp. and *Lelliottia* spp., which were relatively close phylogenetically, with distances of 0.126 and 0.113 between *H. chinensis* and *L. nimipressuralis*, and *H. chinensis* and *L. amnigena*, respectively. The second group comprises strains of the genera *Rahnella* spp., *Serratia* spp., *Klebsiella* spp., *Escherichia* spp. and *Enterobacter* spp. to the genus *Rahnella* spp.. The minimum phylogenetic distance between the first group and *R. aceris* (representing the second group) was 0.209. It was noted that the long horizontal branches of *R. aceris* strain indicate significantly high divergence from the rest of the group. It was also note that the *E. ludwigii* strains (A1 to A13) formed a distinct monophyletic clade with low internal divergence. To see whether the equine *E. ludwigii* strains were genomically related, we added them to a database of 205 *E. ludwigii* genomes from global sources retrieved from NCBI (S2 Fig and S7 Table). The tree generated showed many branches, which means that there is genetic diversity between *E. ludwigii* genomes throughout the world: this allows us to conclude that the *E. ludwigii* A1-A13 in our study are just genomically particularly close.

### Resistome and antibiotic resistance profile

S8 Table presents the resistome raw data, and S9 Table presents raw data obtained from the antimicrobial susceptibility tests. As it can be observed on Fig 2, as group III *Enterobacteriaceae*, the *H. chinensis* and *Enterobacter* spp. strains had chromosomal β-lactamases (*blaACT*). The *L. amnigena* and *R. aceris* strains code for class A penicillinases (*blaLAQ-1* and *blaRAHN* respectively). However, the *L. nimipressuralis* strains were found to possess an acquired *blaCMY* β-lactamase. These enzymes are responsible for natural resistance to amoxicillin in all strains. It has been noted that *E. hormaechei* B2 (ST114) carries acquired β-lactamases such as *blaDHA-1*, *blaOXA-1* and *blaSHV-12*. The acquired β-lactamase *blaTEM-1B* was also observed in *E. hormaechei* B1 (ST171), *E. hormaechei* B4 (ST1644) and *E. hormaechei* B5 (ST1644). The *blaACT* enzyme confers resistance to cefalexin and cefoxitin in 100% of *H. chinensis* (2/2), 100% of *E. hormaechei* (5/5) and 92.3% of *E. ludwigii* (12/13) strains. We found that 100% of *H. chinensis*, *Lelliottia* spp. and *Enterobacter* spp. strains were resistant to the 3rd-generation cephalosporin cefixime, due to β-lactamases, while only *R. aceris* E1 (50% of *R. aceris* strains) was resistant. For the 3rd-generation cephalosporins ceftazidime and ceftriaxone and the 4th-generation cephalosporin cefepime, all strains were susceptible, except for *E. hormaechei*, of which 100% (5/5) were resistant to ceftazidime, 80% (4/5) to ceftriaxone, and 40% (2/5) to cefepime. No strain was resistant to penems (ertapenem, imipenem and meropenem) and 60% of *E. hormaechei* strains (3/5) were resistant to aztreonam.

The *H. chinensis*, *Lelliottia* spp. and *E. ludwigii* strains showed no resistance to aminoglycosides, despite the sporadic presence of certain aminoglycoside-modifying enzymes in some strains. However, the *R. aceris* E2 (50% of *R. aceris* strains) was found to be resistant to amikacin, and 40% of *E. hormaechei* strains were resistant to amikacin and gentamycin.

For quinolones, 100% of *H. chinensis*, *Lelliottia* spp. *R. aceris* and *E. ludwigii* strains were susceptible to ciprofloxacin, levofloxacin and nalidixic acid. *E. hormaechei* B2 (ST114) was resistant to all these antibiotics, and *E. hormaechei* B1 (ST171) was resistant to ciprofloxacin and nalidixic acid. Notably, *E. hormaechei* B2, *E. hormaechei* B4 and *E. hormaechei* B5 were the only strains found to carry quinolone resistance genes, including *aac(6’)-ib_cr*, *qnrB2*, and *qnrB4*.

For other antibiotics tested, i) all strains were resistant to clarithromycin, with the exception of *L. amnigena* D1; ii) We found that only 16.7% (2/12) of *Lelliottia* spp. strains and 7.7% (1/13) of *E. ludwigii* strains were resistant to Fosfomycin, while the *fosA* or *fosA2* genes were found in 100% (2/2) of *H. chinensis* strains, 94.4% (17/18) of *Enterobacter* spp. strains and 100% (6/6) of *L. nimipressuralis* strains; iii) *E. hormaechei* B2 (ST114) and *E. hormaechei* B4 (ST1644) were the only strains to possess at least one chloramphenicol resistance gene and chloramphenicol resistance; iv) similarly, 80% of *E. hormaechei* (4/5) strains were resistant to trimethoprim and cotrimoxazole associated with the presence of resistance genes, and also showed resistance to tigecycline.

Plasmidome analysis revealed the presence of several plasmid families within the strains: IncI, IncF, IncHI, IncR, and Col (Fig 2 and S10 Table). *E. hormaechei* strains were found to be the most frequent plasmid carriers (4/5), with at least two plasmids per strain. The *E. hormaechei* B2 (ST114) strain carries the highest number of plasmids (Col440II, Col(pHAD28), IncHI2, IncR). Fifteen percent of *E. ludwigii* strains carried plasmids (2/13), including *E. ludwigii* A11 carrying five plasmids and *E. ludwigii* A4 carrying two plasmids. Among *Lelliottia* spp., 25% (3/12) of strains carried a single plasmid (IncFII(pECLA) or Col(pHAD28)). *H. chinensis* F1 carried the IncFIB(pHCM2) plasmid, and the *Rahnella spp.* strains showed no plasmids.

### Virulence genes

**Fig 3:**
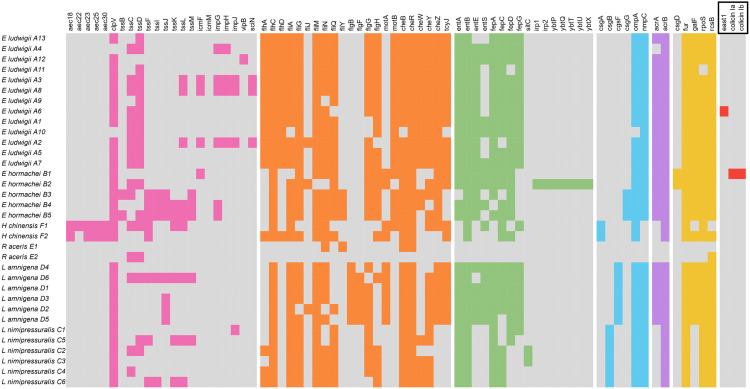
Virulence genes found in equine isolates (coloured squares). Genes involved in the synthesis of T6SS (pink), flagella (orange), competition for environmental nutrients (green), tissue adhesion and invasion (blue), antibiotic efflux (violet), synthesis of transcriptional regulators (yellow) and toxins (red). Boxed genes are plasmid-encoded.

Seven families of virulence factors were found (Fig 3): genes involved in secretion system synthesis, *flagellum* synthesis, competition for nutrients in extracellular environment, tissue adhesion and invasion, antimicrobial resistance, transcriptional regulators and toxin-encoding genes. Genes involved in *flagellum* synthesis were the most widespread among the strains in the collection. By contrast, *east1* and colicin/colicin IB genes encoding toxins were only found in two strains, *E. ludwigii* A6 and *E. hormaechei* B1, respectively. These genes have been acquired and are carried by a plasmid and are not commonly found in *E. hormaechei*. *Enterobacter* spp. have relatively conserved profiles between strains. However, *E. hormaechei* B2 (ST114) strain has additional genes involved in yersiniabactin synthesis (*irp1, irp2, ybtP, ybtQ, ybtT, ybtU and ybtX*). The two strains belonging to the genus *Huaxiibacter* spp. also exhibit similar arsenal of virulence genes as other genera. They both possessed others genes (*aec18*, *aec23*, *aec25*, *aec30*) involved in the synthesis of the T6SS. In contrast, they lacked certain genes associated with the same function (*icm*, *imp*, *vipB*, and *sciN*) that were present in some *Enterobacter* spp. They are also the only ones to carry the *csgA* gene, which encodes the major subunit of *curli* (adhesin). *R. aceris* E1 and E2 strains belonged to the genus with the fewest virulence factors. *R. aceris* E1 harbored only genes related to flagellar synthesis (*fliN, fliY, cheB*, and *cheR*), while *R. aceris* E2 possessed only two genes involved in T6SS synthesis, along with the transcriptional regulator *rscB*, which plays a role in regulating this process. For the genus *Lelliottia* spp., the virulome was more similar to that of the genus *Enterobacter*. However, the genes involved in *flagella* synthesis are less diversified. *L. amnigena* D6 was the strain with the most genes involved in this process (n = 9). Divergences appeared between the species *L. amnigena* and *L. nimipressuralis*. Indeed, *L. amnigena* possesses the *csgF* gene, while *L. nimipressuralis* has the *csgB* gene (coding for adhesin proteins). *L. nimipressuralis* strains lacked certain genes *L. amnigena* strains had, such as *flgB*, *cheWZ*, *tcyJ* (*flagella* synthesis), *entE* and *entS* (enterobactin), *acrA* (antibiotic efflux pump), and *galF* (transcriptional regulator).

## Discussion

This study allowed the analysis of a unique collection of *Enterobacteriaceae* strains obtained from horse necropsies and collected over an extended period (1997–2020). As these strains were suspected to be implicated in equine mortality, the characterization of their species, virulome, resistome, and antimicrobial resistance profiles is particular importance, especially within the One Health framework.

We first aimed to confirm the identification of the strains using mass spectrometry (MALDI-TOF), a technique commonly employed for microorganism identification in clinical microbiology. As MALDI-TOF failed to identify nine of the 34 strains, we used rMLST to complete and confirm the identification of strains at both the genus and species levels. This approach thus allowed us to identify several genera within the collection, including *Enterobacter* (previously identified using the API20E gallery), as well as *Lelliottia* spp*.*, *Huaxiibacter* spp*.*, and *Rahnella* spp*.*. As demonstrated by a study conducted in Denmark comparing identification methods, MALDI-TOF proves useful for the accurate characterization of commonly encountered strains such as *K. pneumoniae* and *Staphylococcus aureus*. However, this technique is less discriminating for strains within the *E. cloacae* complex and struggles to accurately identify less well-characterized strains at the species [28]. The unprecise assignments by MALDI-TOF compared to rMLST (26.5% of common identification) was notably due to the recent reclassification following advancements in sequencing techniques. For example, *Enterobacter nimipressuralis* and *Enterobacter amnigena* were reclassified in 2013 as *L. nimipressuralis* and *L. amnigena* based on sequencing of the *gyrB*, *rpoB*, *infB*, and *atpD* genes [9]. Similarly, the genus *Huaxiibacter* was newly described in 2022 [29]. The genus *Rahnella* differs somewhat; it was first proposed in 1979 and was reclassified in 2016 as belonging to the *Yersiniaceae* family instead of the *Enterobacteriaceae* family [30]. In comparison to the identifications made by rMLST, it was observed that the genera *Lelliottia* spp. and *Rahnella* spp. were consistently identified by MALDI-TOF, although species-level identification was not achieved. Conversely, 86% of *Enterobacter* spp. identified by MALDI-TOF were subsequently confirmed by rMLST, while 14% were reclassified by rMLST as belonging to the genus *Huaxiibacter*. In addition, the difficulty in identifying the genera *Rahnella* spp., *Lelliottia* spp., and *Huaxiibacter* spp. using MALDI-TOF is explained by the MBT 8468 MPS database (Bruker) employed, which contains only a limited number of reference spectra for these genera (seven spectra for *Rahnella* spp. and *Lelliottia* spp*.*, and none for *Huaxiibacter* spp.).Taken together, of the two identification methods tested, WGS combined with rMLST analysis proved to be the most discriminating and particularly useful for population-level studies. Furthermore, identification by MALDI-TOF depends on database used: as ours is tailored specifically to human medicine, it may be inappropriate for the caracterisation of less well characterized species. However, MALDI-TOF remains a fully applicable method, especially when rapid diagnosis is required, as it provides a reasonably accurate genus-level identification, particularly for genera known to pose potential challenges.

MLST analysis of *Enterobacter* strains revealed a high diversity of STs, suggesting a lack of close genomic relatedness among the strains. ST1629 (*E. ludwigii A4 and A13*) and ST1644 (*E. hormaechei B4 and B5*) were each detected in two strains originating from different locations, indicating that these occurrences are unlikely to result from clonal dissemination. Moreover, these STs have not been previously described in the literature. Of particular note, two other *E. hormaechei* STs, ST114 and ST171, were also identified within the collection. These ST had been reported multiple times in various studies and is recognized as an international high-risk clone. ST171 was documented during an outbreak in a New York hospital, where it accounted for 50% of identified STs in 2014 [31], and its prevalence reached 28% of *Enterobacter* spp. in a hospital in Shandong, China, in 2023 [32]. This clone has also been detected in environments in contact with animals, such as zoological facilities in 2023 [33], and in companion cats in 2024 [34]. The *E. hormaechei* B1 ST171 isolate also carries the IncFIB plasmid, which has been identified in ST171 strains producing the KPC-4 carbapenemase [33]. The presence of IncFIB(K), IncFIB(pHCM2) and IncI1-I(Alpha) plasmids in *E. hormaechei* B1 could be responsible for carrying the acquired resistance genes *blaCMY-2 and bla-TEM-1B*. ST114 has also been frequently identified in both humans and animals. Its presence in animals is thought to reflect anthropogenic contamination of the environment [35]. ST114 strains recovered worldwide are extended-spectrum β-lactamase (ESBL) producers, commonly carrying *blaOXA*, *blaSHV*, *blaCTX-M* and *blaTEM* [36,37], and exhibit extensive resistance to 3rd-generation cephalosporins, like our isolate. ST114 is frequently associated with the IncHI2 plasmid [35], which was also detected in our isolate. This plasmid is typically responsible for ESBL gene carriage in most strains. However, due to the absence of complete plasmid sequencing in our study, we cannot confirm whether *blaDHA-1 blaOXA-1*, or *blaSHV-12* (found in *E. hormaechei* ST114) are located on this plasmid. These various cases illustrate the ability of bacteria to become clonal population and to acquire mobile genetic elements carrying antibiotic resistance genes conferring multidrug resistance. However, *Enterobacter* spp. harbored the highest number of resistance genes and showed resistance to at least three classes of antibiotics in 26% of cases in 2016 and 52% in 2019 [3,10]. In our study, 22% of the strains from our collection were also resistant to at least three antibiotic classes, particularly *E. hormaechei* strains isolated between 2006 and 2017. Among the *E. hormaechei* strains, 80% (4/5) showed resistance to at least three classes of antibiotics, excluding β-lactams (including aminoglycosides, macrolides, quinolones, chloramphenicol, sulfonamides, and tetracyclines). For *E. ludwigii*, 8% (1/13) of strains were resistant to at least two classes of antibiotics. However, we observed one isolate with a high resistance potential: the *E. hormaechei* B2 isolate, corresponding to ST114. As this sequence type has been associated with human outbreaks, its presence in horses could result from human-to-animal transmission. However, no documented cases support this type of transmission, whereas animal-to-human transmission (zoonosis) is frequently reported [38].

Strains belonging to the same ST may still exhibit differences in their resistome and phenotypic profiles, likely due to environmental differences and genomic plasticity. The genus *Enterobacter* spp. displays a richer virulome, indicative of higher virulence and potential pathogenicity. For example, we detected the presence of the enteroaggregative heat-stable enterotoxin 1 (EAST1) and colicin, synthesized by *E. ludwigii* A6 (ST2797) and *E. hormaechei* B1 (ST171), respectively. The EAST1 was originally characterized in *E. coli* and is known to induce intestinal water secretion, leading to diarrhea in both humans and animals [39]. Colicin is a bacteriocin with bactericidal activity [40], providing a competitive advantage. It acts by forming pores in the bacterial cell membrane [41]. Its gene is carried by high-molecular-weight plasmids of the heterogeneous IncFI group [42]. Notably, the *E. hormaechei* B1 harbors two plasmids belonging to this group, suggesting that the colicin gene may be carried by one of them. We also observed the specific presence of the *irp1*, *irp2*, and *ybtPQTUX* genes in *E. hormaechei* B2, which are involved in iron chelation. The *irp1* and *irp2* genes are responsible for the synthesis of HMWP1 and HMWP2 (high-molecular-weight proteins 1 and 2) [43], while the *ybt* genes are involved in the transport of the yersiniabactin–Fe3+ complex, as well as in the regulation, maturation, resistance to self-intoxication, and recycling of yersiniabactin. In addition to providing a competitive advantage, yersiniabactin contributes to oxidative stress tolerance [44] and may serve as a marker of increased pathogenic potential. Genes involved in motility and adhesion are also widely distributed across the different genera. Motility and adhesion are key factors for colonization, environmental persistence, and virulence, making them central elements in pathogenicity. Genes associated with the T6SS are also widely found across the four genera. This secretion system is broadly distributed among Gram-negative bacteria and can be highly variable, particularly within the genus *Enterobacter* spp. [16]. The T6SS is involved in metal acquisition (Zn2+ and Mn2+), the response to stress induced by reactive oxygen species (ROS), and acidic pH conditions such as those encountered within macrophages [45]. Additionally, T6SS contributes to virulence by acting as an injector of effectors into eukaryotic cells. It has been shown to induce hemolytic activity and facilitate adhesion to epithelial cells during infection [16].

The genera *Lelliottia* spp., *Huaxiibacter* spp and *Rahnella* spp. are sporadically implicated in human infections such as septicemia in drug addicts or premature newborns [46,47], septic shock in immunocompetent individuals [48] or pyonephrosis [49]. However, no data have yet been published on its involvement in equine infections/mortality and limited data exist on their antimicrobial susceptibility profiles. Based on the collection, these different genera would account for a prevalence of presence in equine necropsy samples of 47%, while *Enterobacter* spp. would account for a prevalence of 53%. The genera *Rahnella* spp., *Huaxiibacter* spp. and *Lelliottia* spp. exhibited distinct resistomes and antibiotic resistance profiles. For example, *Rahnella* spp. and *Lelliottia* spp. have their own chromosomal β-lactamases (*blaRAHN* and *blaLAQ-1*, respectively). Overall, we observed that 50% (1/2) of *R. aceris* strains and 16.7% (2/12) of *Lelliottia* spp. strains were resistant to at least two classes of antibiotics (excluding β-lactams). These resistances involved macrolides (clarithromycin), aminoglycosides (amikacin and gentamicin), or fosfomycin. We also observed that *H. chinensis* strains were resistant only to certain β-lactams and clarithromycin, making these strains the least resistant to the antibiotics tested. Despite a limited resistome (with few or no known antibiotic resistance genes), *Lelliottia* spp., *R. aceris*, and *H. chinensis* nonetheless displayed antibiotic resistance, suggesting the involvement of alternative mechanisms such as efflux pumps, reduced membrane permeability, or chromosomal mutations. Conversely, other genotype–phenotype discrepancies were also observed. The presence of resistance genes such as *fosA2*, *aac(6’)-Ib-cr*, and *qnrB2* in certain strains was not consistently associated with a resistant phenotype, suggesting a possible role of transcriptional regulation, conditional gene expression, or inactivating mutations. These findings highlight the importance of interpreting genomic data with caution and always correlating it with phenotypic evidence. Moreover, although the genera *Huaxiibacter* spp., *Rahnella* spp., and *Lelliottia* spp. are only sporadically associated with human clinical infections [46–49], they are predominantly found in the environment. Their presence in necropsy samples may suggest that they were part of the equine microbiota, possibly due to contamination of drinking water, for example. The potentially environmental origin of these strains could be at the root of their low resistance to antimicrobials and the absence of acquired resistance genes (low selection pressure). The presence of plasmids in some of these strains may then have other roles, such as promoting resistance to environmental stresses (tolerance, degradation, detoxification of heavy metals, for example) [50].

We observed that virulence profiles vary across genera and species. However, the same categories of genes—those involved in T6SS, flagellar synthesis, environmental resource competition, adhesion, AcrAB efflux pump synthesis, and transcriptional regulators— have been found in *Lelliottia* spp. and *Huaxiibacter* spp.. The *R. aceris* strains harbor a very limited virulome, suggesting either low pathogenic potential or the presence of virulence factors that have not yet been characterized.

## Conclusion

This study, conducted on a unique collection of *Enterobacteriaceae* isolated from necropsied horses in Normandy over more than two decades, highlights an unexpected taxonomic and genomic diversity. Compared to MALDI-TOF, rMLST proved to be the most relevant approach for the accurate identification of strains, revealing the presence of recently described or reclassified genera such as *Lelliottia* spp*.*, *Huaxiibacter* spp*.*, and *Rahnella* spp*.*. This diversity underscores the limitations of conventional identification methods and reinforces the value of the whole-genome sequencing as a reference tool in veterinary microbiology. Genomic analysis revealed substantial heterogeneity in STs of *Enterobacter* spp., with no evidence of clonal transmission, yet the detection of high-risk clones known in human medicine (*E. hormaechei* ST114 and ST171) raises questions about potential interactions between animal, environmental, and human microbiota, within a One Health framework. The presence of antibiotic acquired resistance genes and plasmids suggests that *E. hormaechei* strains may act as reservoirs of mobilizable resistance determinants. In parallel, the variability of virulence profiles across genera (from highly pathogenic strains in *Enterobacter* spp*.* to less virulent genus like *Rahnella* spp*.*) reflects an evolutionary dynamic that remains poorly understood. Nevertheless, the detection of genes involved in T6SS, adhesion, motility, or iron metabolism suggests that these environmental strains could become opportunistic under certain conditions and potentially contributing to equine infections.

## Supporting information

S1 Table Characteristics of isolates from necropsied equids. (XLSX)

S2 Table Antibiotics tested for susceptibility testing on Mueller-Hinton agar medium. (XLSX)

S3 Table *Enterobacteriaceae* reference genomes used in the phylogenetic study of the equine collection. (XLSX)

S4 Table Identification of isolates using either the MALDI-TOF method or WGS/rMLST. ND = Not determined.
(XLSX)

S5 Table Analysis of distances between genomes using the MASH method. (XLSX)

S6 Table Comparative statistical analysis (Fisher’s exact test). (XLSX)

S7 Table *Enterobacter ludwigii* worldwide genomes used for the phylogenetic study of the *Enterobacter ludwigii* Normandy’s isolates. (XLSX)

S8 Table Resistome characterization using ResFinder with a threshold of 90% identity and coverage. (XLSX)

S9 Table Antimicrobial susceptibility tests (R = resistant; S = sensitive). (XLSX)

S10 Table Plasmids identified in the isolates, as determined by PlasmidFinder using a minimum identity threshold of 0.8 and a coverage threshold of 0.7. (XLSX)

S1 Fig Aspect of *Enterobacter* spp. (A), *Lelliottia* spp. (B), *Huaxiibacter* spp. (C), *Rahnella* spp. (D) on CPSE agar.
(TIF)

S2 Fig Comparative phylogenetic tree of equine *E. ludwigii* (from this study, in red) and 205 *E. ludwigii* genomes available from the NCBI database. (TIF)
