# Supplementary figures and images for: Genomic epidemiology of strains currently and formerly classified as Enterobacter spp. recovered from equine necropsy samples
Source: PLoS One. 2025 Nov 13;20(11):e0333701. doi: 10.1371/journal.pone.0333701 (PMC12614608; doi:10.1371/journal.pone.0333701)

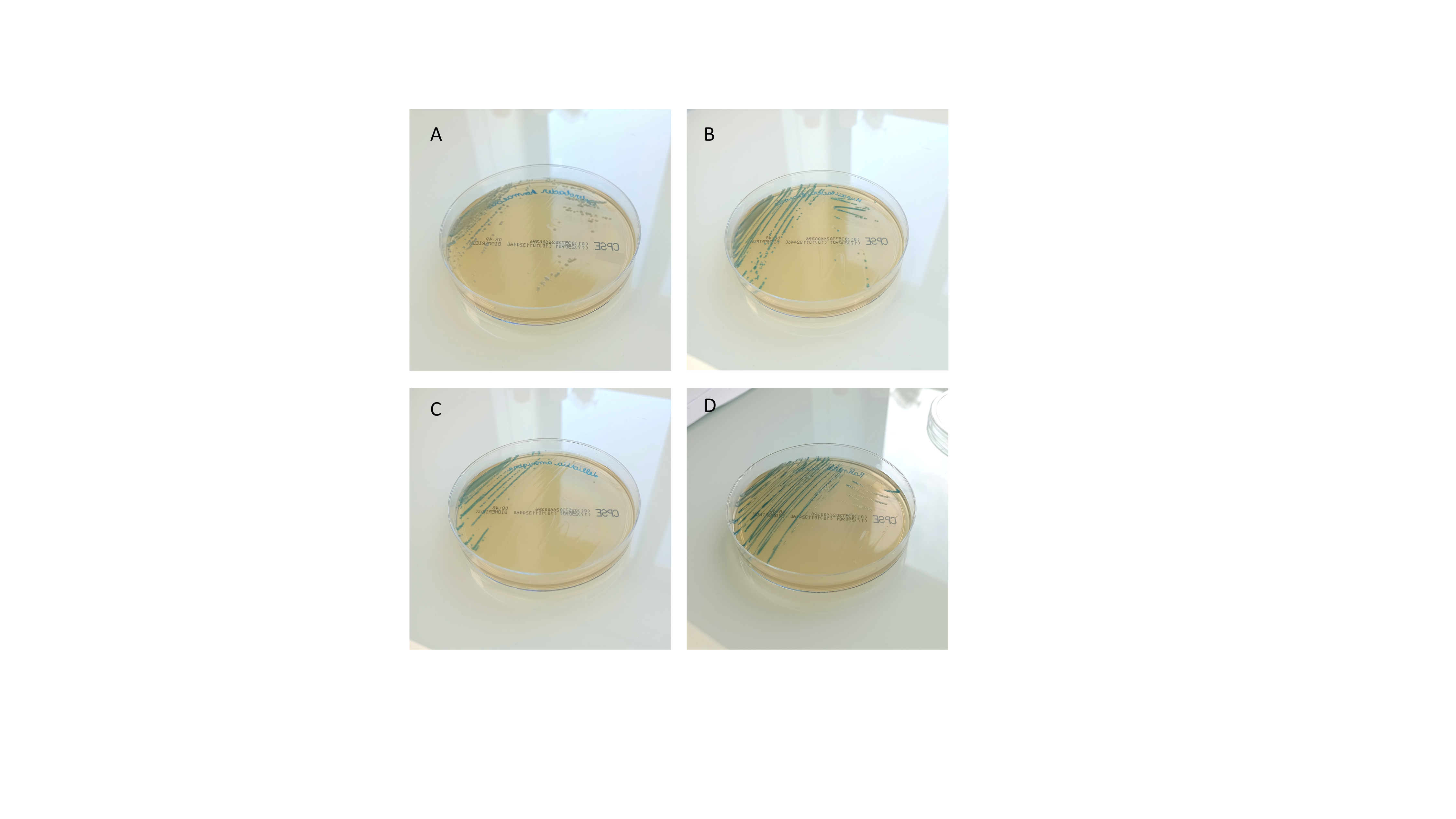

Supplement: S1 Fig — (A), Lelliottia spp. (B), Huaxiibacter spp. (C), Rahnella spp. (D) on CPSE agar. (TIF) [file pone.0333701.s011.tif]

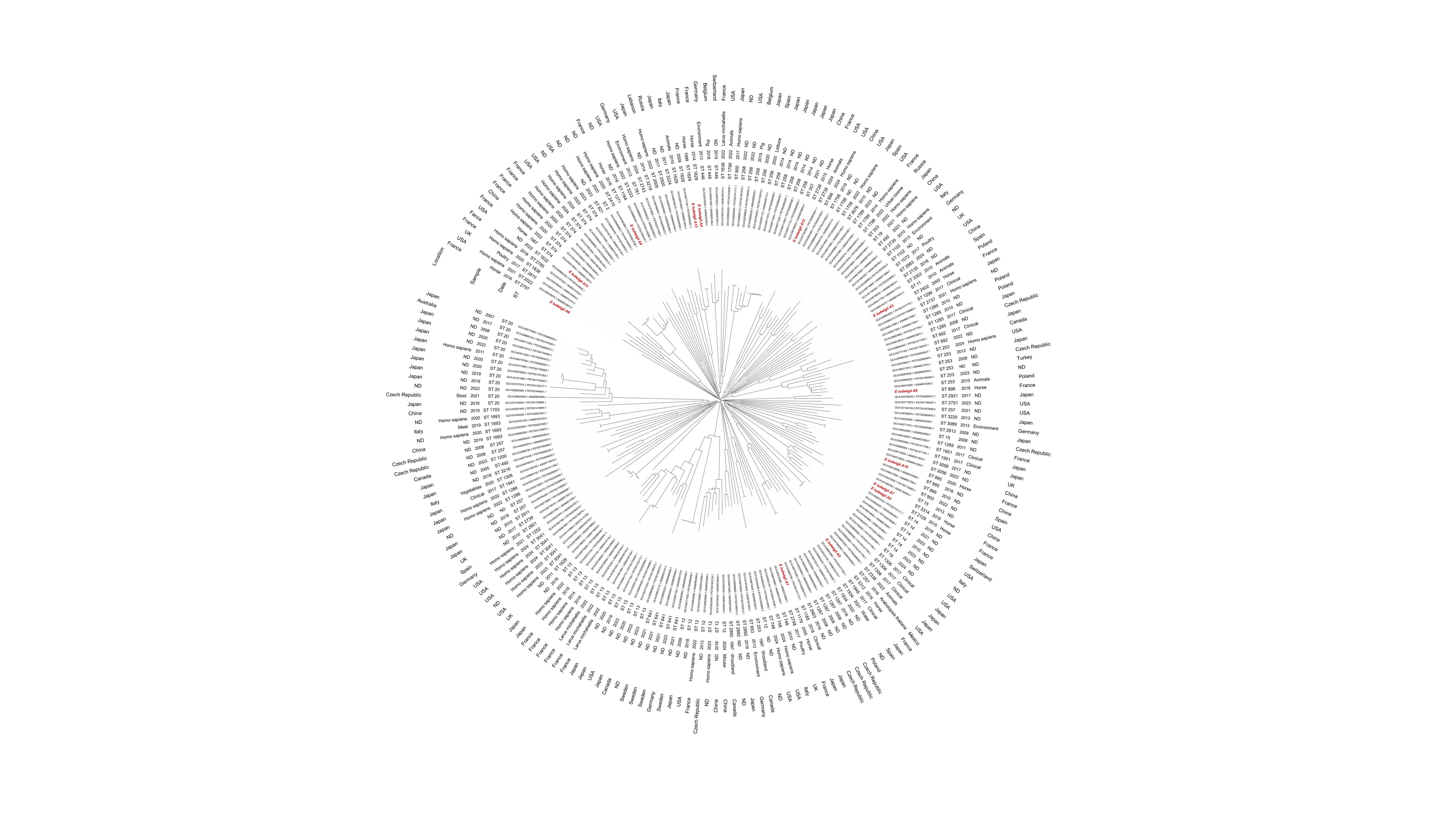

Supplement: S2 Fig — (TIF) [file pone.0333701.s012.tif]
